# Unraveling Pancreatic Cancer: Epidemiology, Risk Factors, and Global Trends

**DOI:** 10.7759/cureus.72816

**Published:** 2024-11-01

**Authors:** Rana Muhammad Anss Bin Qadir, Musab Bin Umair, Umar Bin Tariq, Arslan Ahmad, Wajeeha Kiran, M Hasaan Shahid

**Affiliations:** 1 Surgery, Queen’s Hospital, Romford, GBR; 2 Surgery, Meriden Hospital, Conventry, GBR; 3 General Surgery, Southmead Hospital Bristol, North Bristol NHS Trust, Bristol, GBR; 4 Emergency Medicine, Weston General Hospital, University Hospitals Bristol and Weston NHS Foundation Trust, Weston-super-Mare, GBR; 5 Trauma and Orthopaedics, Morriston Hospital, Swansea, GBR; 6 Surgery, Glangwili General Hospital, Carmarthen, GBR

**Keywords:** brca mutations, chronic pancreatitis, neoadjuvant therapy, pancreatic cancer, pancreatic ductal adenocarcinoma (pdac)

## Abstract

Pancreatic cancer is one of the most lethal malignancies, characterized by late diagnosis, rapid progression, and limited treatment options. This literature review comprehensively examines the epidemiology, risk factors, diagnostic challenges, treatment modalities, and prognosis of pancreatic cancer. It highlights the global disparities in incidence and outcomes, exploring the influence of socioeconomic, environmental, and genetic factors on disease progression. In addition, this review discusses recent advancements in diagnostic tools and treatment strategies, identifying gaps in current research and clinical practices. The synthesis aims to inform future research directions and policy-making efforts to reduce the global burden of pancreatic cancer and improve patient outcomes.

## Introduction and background

Pancreatic cancer represents a significant challenge in oncology due to its aggressive nature and poor prognosis. It primarily manifests as pancreatic ductal adenocarcinoma (PDAC), which accounts for over 90% of cases [1]. The disease is often asymptomatic in its early stages, leading to diagnosis at advanced stages when curative treatment is rarely possible. This late-stage diagnosis contributes to the high mortality rate, making pancreatic cancer the seventh leading cause of cancer-related deaths worldwide, despite being only the 10th most common cancer. The disease's five-year survival rate remains below 10%, underscoring the need for improved detection and treatment strategies [2].

This review explores the epidemiology, risk factors, diagnostic challenges, and treatment options for pancreatic cancer, focusing on the disparities that influence outcomes. By synthesizing current research and identifying gaps in knowledge, this review aims to provide a foundation for future studies and policy initiatives to combat this devastating disease.

## Review

Epidemiology of pancreatic cancer

Global Incidence and Mortality Trends

Pancreatic cancer is a global health concern with significant variation in incidence and mortality rates across different regions. In 2018, approximately 458,918 new cases were reported worldwide [2]. The highest incidence rates are found in Europe and North America, with countries like Hungary reporting age-standardized rates (ASRs) as high as 11.2 per 100,000. By contrast, Southeast Asia has some of the lowest incidence rates, around 1.6 per 100,000. These regional differences reflect variations in risk factor prevalence, healthcare access, and diagnostic capabilities [3].

Mortality trends closely mirror incidence rates, with pancreatic cancer being the seventh leading cause of cancer-related deaths globally. In high-income countries, mortality rates have either stabilized or increased slightly, while in low- and middle-income countries, mortality continues to rise. This increase is attributed to an aging population, changes in lifestyle factors, and improvements in diagnosis that have led to more reported cases [1,4].

Age, Gender, and Ethnic Disparities

Age is a significant risk factor for pancreatic cancer, with incidence increasing sharply after the age of 50 [5]. The majority of cases are diagnosed in individuals aged 65 and older [6]. Gender also plays a role, with men generally having higher incidence and mortality rates than women [7]. This difference is partly attributed to higher smoking rates among men and occupational exposures that are more common in male-dominated industries. However, recent studies have shown a concerning rise in pancreatic cancer incidence among younger women, particularly those of Hispanic and Black backgrounds. This trend suggests that factors beyond traditional risk markers may be at play, necessitating further investigation [8].

Ethnic disparities are particularly pronounced in pancreatic cancer. In the United States, African Americans have the highest incidence and mortality rates, nearly 30% higher than those of White Americans. This disparity is likely due to a combination of genetic predispositions, lifestyle factors, and socioeconomic barriers to healthcare. Other ethnic groups, including Native Americans and Hispanics, also face higher risks, reflecting the complex interplay of genetics, environment, and access to care [9].

Socioeconomic Disparities

Socioeconomic status (SES) significantly influences pancreatic cancer outcomes. Individuals with lower SES are more likely to be diagnosed at later stages, have limited access to treatment, and experience worse overall survival [10]. These disparities persist even in countries with universal healthcare systems, such as the Netherlands, where lower SES is associated with reduced survival rates. Factors contributing to these disparities include lower health literacy, delayed diagnosis, and reduced access to specialized care. Addressing these socioeconomic barriers is crucial for improving outcomes and achieving equity in pancreatic cancer care [11].

Risk factors for pancreatic cancer

Genetic Predispositions

Genetic factors play a crucial role in the development of pancreatic cancer. Approximately 10% of cases are attributed to hereditary factors, with mutations in genes such as BRCA1, BRCA2, PALB2, and CDKN2A being associated with increased risk [12]. Familial pancreatic cancer, where multiple family members are affected, is often linked to these genetic mutations. Lynch syndrome and hereditary pancreatitis are other inherited conditions that significantly elevate the risk of developing pancreatic cancer [13].

The discovery of these genetic links has opened new avenues for risk assessment and early detection. Individuals with a family history of pancreatic cancer or related genetic syndromes may benefit from regular screening and genetic counseling. However, the challenge remains in translating these findings into effective prevention and treatment strategies for the broader population [14].

Lifestyle and Environmental Factors

Lifestyle factors are major contributors to pancreatic cancer risk. Smoking is the most well-established risk factor, accounting for approximately 20-25% of cases. Smokers are twice as likely to develop pancreatic cancer as non-smokers, and the risk remains elevated for up to 20 years after smoking cessation [15]. Alcohol consumption, particularly in large quantities, is another significant risk factor, especially when combined with smoking [16].

Dietary habits also play a crucial role in pancreatic cancer risk. Diets high in red and processed meats, saturated fats, and sugar have been linked to increased risk, while diets rich in fruits, vegetables, and whole grains may offer protective benefits. Recent studies suggest that the Mediterranean diet, characterized by a high intake of fruits, vegetables, nuts, and olive oil, may reduce pancreatic cancer risk [17]. Obesity is another important risk factor, with individuals who are obese having a 20% higher risk of developing the disease. The relationship between obesity and pancreatic cancer is partly mediated by insulin resistance, chronic inflammation, and changes in adipokines, which are bioactive molecules produced by adipose tissue [18].

Diabetes Mellitus and Chronic Pancreatitis

Diabetes mellitus, particularly type 2 diabetes, is closely associated with pancreatic cancer. Patients with long-standing diabetes have a 50-100% increased risk of developing pancreatic cancer compared to non-diabetics [19]. Interestingly, new-onset diabetes can be both a risk factor and an early symptom of pancreatic cancer. The bidirectional relationship between diabetes and pancreatic cancer is complex, with hyperglycemia, insulin resistance, and chronic inflammation contributing to carcinogenesis [20].

Chronic pancreatitis, a condition characterized by long-term inflammation of the pancreas, is another significant risk factor. The persistent inflammation leads to fibrosis and cellular changes that can predispose individuals to cancer [21]. Hereditary pancreatitis, caused by mutations in the PRSS1 gene, carries an even higher risk, with affected individuals having a 40-55% lifetime risk of developing pancreatic cancer [12].

Occupational and Environmental Exposures

Occupational exposures to certain chemicals and heavy metals have been implicated in pancreatic cancer risk. Workers in industries such as metalworking, rubber manufacturing, and pesticide production may be at higher risk due to prolonged exposure to carcinogens. For instance, chlorinated hydrocarbons, used in metal cleaning and degreasing, have been linked to increased pancreatic cancer risk. In addition, exposure to asbestos, which is known for causing mesothelioma, has also been associated with pancreatic cancer in some studies [22].

Environmental exposures, such as air pollution and contaminated water, are emerging risk factors that warrant further investigation. Studies have shown that individuals living in areas with high levels of air pollution, particularly fine particulate matter (PM2.5), have a higher risk of developing pancreatic cancer. These findings underscore the need for public health interventions to reduce environmental exposures and mitigate their impact on cancer risk [23,24].

Diagnosis of pancreatic cancer

Challenges in Early Detection

One of the biggest challenges in pancreatic cancer management is early detection. The disease often presents with non-specific symptoms such as abdominal pain, weight loss, and jaundice, which are typically indicative of advanced disease. By the time these symptoms appear, the cancer has usually metastasized, making curative treatment difficult [25].

Currently, there are no reliable screening tests for the general population. High-risk individuals, such as those with a family history of pancreatic cancer or known genetic mutations, may undergo regular surveillance using imaging techniques like MRI or endoscopic ultrasound (EUS). However, these methods are invasive, expensive, and not widely available [26].

Imaging Techniques

Imaging plays a crucial role in the diagnosis and staging of pancreatic cancer. The most commonly used modalities are computed tomography (CT) scans, magnetic resonance imaging (MRI), and EUS. CT scans are often the first-line imaging test due to their availability and effectiveness in detecting pancreatic masses and assessing their resectability. Multiphase contrast-enhanced CT, also known as pancreatic protocol CT, provides detailed images of the pancreas and surrounding structures, helping to evaluate the extent of the tumor and its relationship with major blood vessels [26,27].

MRI offers superior soft tissue contrast compared to CT and is particularly useful in detecting small tumors and assessing liver metastases. Diffusion-weighted imaging (DWI) is a specialized MRI technique that enhances the detection of small lesions by measuring the movement of water molecules within tissues. EUS, which involves the use of an endoscope with an ultrasound probe, allows for high-resolution imaging of the pancreas and surrounding structures. It is especially useful for obtaining tissue samples through fine-needle aspiration (FNA) for histopathological analysis [28].

Biomarkers and Molecular Diagnostics

Biomarkers are substances that can be measured in the blood, urine, or tissues to indicate the presence of cancer. The most commonly used biomarker for pancreatic cancer is CA 19-9, a carbohydrate antigen that is elevated in about 80% of patients with pancreatic cancer. However, CA 19-9 is not specific to pancreatic cancer and can be elevated in other conditions such as cholangitis, cirrhosis, and other gastrointestinal cancers. Its utility is primarily in monitoring treatment response and detecting disease recurrence rather than in early diagnosis [29].

Advances in molecular diagnostics are paving the way for the development of more sensitive and specific biomarkers. Liquid biopsies, which detect circulating tumor DNA (ctDNA) or circulating tumor cells (CTCs) in the blood, hold promise for early detection and monitoring of pancreatic cancer. Studies have shown that ctDNA can be detected in a significant proportion of patients with pancreatic cancer, even in the early stages. However, these techniques are still in the experimental stage and require further validation before they can be implemented in clinical practice [30,31].

Trends in Five-Year Survival by Cancer Staging

**Figure 1:**
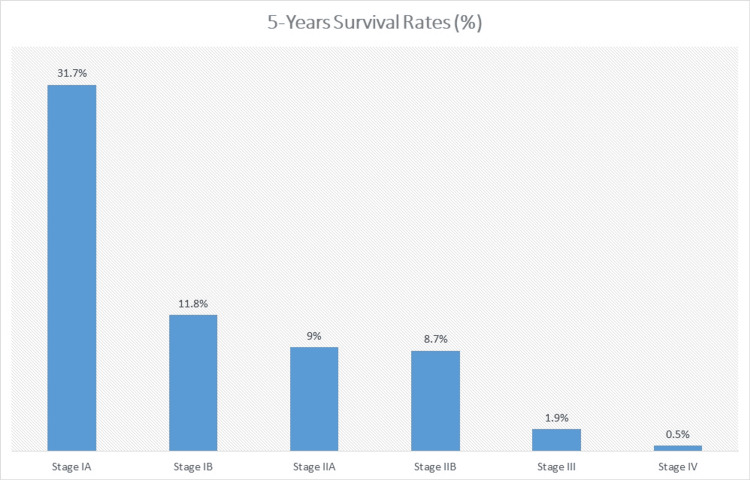
Five-year survival rates for pancreatic cancer based on its American Joint Committee on Cancer (AJCC) stage This graph was created by one of the authors using data from the cited article.

Figure 1 demonstrates five-year survival rates for pancreatic cancer based on its American Joint Committee on Cancer (AJCC) stage. These percentages are influenced by factors such as tumor biology, patient health, and the availability of a comprehensive, multidisciplinary treatment approach [32].

Surgical Interventions

Surgery remains the only potentially curative treatment for pancreatic cancer, but only 15-20% of patients are eligible for resection at the time of diagnosis due to the advanced stage of the disease. The most common surgical procedure is the pancreaticoduodenectomy, also known as the Whipple procedure, which involves the removal of the head of the pancreas, the duodenum, part of the bile duct, the gallbladder, and sometimes part of the stomach. This complex procedure is associated with significant morbidity and mortality, but in experienced centers, the mortality rate has decreased to less than 5% [33,34].

For tumors located in the body or tail of the pancreas, a distal pancreatectomy is performed, often along with splenectomy. Total pancreatectomy, which involves the removal of the entire pancreas, is rarely performed due to the significant impact on the quality of life, including the need for lifelong insulin and enzyme replacement therapy [35,36].

Adjuvant and Neoadjuvant Therapies

Adjuvant chemotherapy is standard practice following surgical resection to eliminate microscopic residual disease and improve survival outcomes. Gemcitabine-based chemotherapy has been the standard of care for many years, but more recent trials have shown that combination regimens such as FOLFIRINOX (a combination of fluorouracil, leucovorin, irinotecan, and oxaliplatin) offer superior survival benefits, albeit with increased toxicity [37].

Neoadjuvant therapy, administered before surgery, is increasingly being used for patients with borderline resectable or locally advanced pancreatic cancer. The rationale behind neoadjuvant therapy is to downstage the tumor, making it more likely to be resectable, and to address micrometastatic disease early [38]. Studies have shown that neoadjuvant FOLFIRINOX or gemcitabine plus nab-paclitaxel can lead to higher rates of margin-negative resection and improved overall survival compared to upfront surgery [39].

Chemotherapy and Radiation Therapy

For patients with metastatic or unresectable pancreatic cancer, chemotherapy remains the mainstay of treatment. FOLFIRINOX and gemcitabine plus nab-paclitaxel are the most commonly used regimens, with FOLFIRINOX generally reserved for patients with good performance status due to its higher toxicity profile. These regimens have improved median survival to approximately 11-12 months, but the prognosis remains poor overall [40].

Radiation therapy is used in selected cases, primarily for locally advanced diseases where the tumor is unresectable but has not metastasized. It is often combined with chemotherapy (chemoradiation) to enhance the therapeutic effect [41]. Stereotactic body radiation therapy (SBRT) is an emerging technique that delivers high doses of radiation over a few sessions with minimal exposure to surrounding tissues. SBRT has shown promise in controlling local tumor growth and improving the quality of life in patients with locally advanced pancreatic cancer [42].

Emerging Treatments and Clinical Trials

The treatment landscape for pancreatic cancer is evolving with the introduction of targeted therapies and immunotherapies. Targeted therapies focus on specific molecular alterations in the tumor, such as mutations in KRAS, BRCA, and other genes. PARP inhibitors, which target BRCA-mutated tumors, have shown efficacy in a subset of patients with pancreatic cancer, leading to the approval of olaparib for maintenance therapy in patients with germline BRCA mutations [43].

Immunotherapy, which has revolutionized the treatment of other cancers, has had limited success in pancreatic cancer due to the immunosuppressive tumor microenvironment. However, combination strategies that include immune checkpoint inhibitors, vaccines, and adoptive cell therapies are being actively investigated in clinical trials. The results of these studies are eagerly awaited, as they hold the potential to significantly improve outcomes for patients with this challenging disease [44].

Prognostic Factors

The prognosis for pancreatic cancer remains grim, with a five-year survival rate of less than 10%. The stage at diagnosis is the most important determinant of survival, with patients diagnosed at an early stage having a significantly better prognosis [45]. Tumor biology, including genetic mutations and molecular subtypes, also plays a critical role in determining outcomes. For example, patients with tumors harboring BRCA mutations may respond better to platinum-based chemotherapy or PARP inhibitors [46].

Other prognostic factors include the patient's performance status, the presence of comorbidities, and the response to initial treatment. Despite advances in treatment, most patients eventually experience disease recurrence, underscoring the need for more effective systemic therapies [47].

Importance of Multidisciplinary Care

Given the complexity of pancreatic cancer, a multidisciplinary approach is essential for optimal patient management. This approach involves collaboration among surgeons, medical oncologists, radiation oncologists, gastroenterologists, radiologists, and palliative care specialists. Multidisciplinary tumor boards, where cases are reviewed by a team of specialists, have been shown to improve outcomes by ensuring that patients receive the most appropriate and individualized care [48].

## Conclusions

Pancreatic cancer remains one of the most challenging malignancies to treat, with high mortality and limited therapeutic options. While advancements in surgery, chemotherapy, and emerging therapies have improved outcomes for some patients, the overall prognosis remains poor. Early detection is critical but remains elusive, necessitating further research into novel diagnostic tools and biomarkers. Addressing the global disparities in pancreatic cancer care, improving access to treatment, and advancing our understanding of the disease's molecular underpinnings are essential steps toward reducing the burden of this devastating disease.

Continued efforts in research, clinical trials, and policy-making are needed to develop more effective strategies for prevention, early detection, and treatment. By integrating these advancements with a multidisciplinary approach to care, we can hope to improve the survival and quality of life for patients with pancreatic cancer.
